# Connected partner-switches control the life style of *Pseudomonas aeruginosa* through RpoS regulation

**DOI:** 10.1038/s41598-019-42653-5

**Published:** 2019-04-24

**Authors:** Sophie Bouillet, Moly Ba, Laetitia Houot, Chantal Iobbi-Nivol, Christophe Bordi

**Affiliations:** 1 Aix Marseille Univ, CNRS,IMM, BIP, Marseille, France; 2 Aix Marseille Univ, CNRS, IMM, LISM, Marseille, France

**Keywords:** Bacterial genetics, Biofilms

## Abstract

Biofilm formation is a complex process resulting from the action of imbricated pathways in response to environmental cues. In this study, we showed that biofilm biogenesis in the opportunistic pathogen *Pseudomonas aeruginosa* depends on the availability of RpoS, the sigma factor regulating the general stress response in bacteria. Moreover, it was demonstrated that RpoS is post-translationally regulated by the HsbR-HsbA partner switching system as has been demonstrated for its CrsR-CrsA homolog in *Shewanella oneidensis*. Finally, it was established that HsbA, the anti-sigma factor antagonist, has a pivotal role depending on its phosphorylation state since it binds HsbR, the response regulator, when phosphorylated and FlgM, the anti-sigma factor of FliA, when non-phosphorylated. The phosphorylation state of HsbA thus drives the switch between the sessile and planktonic way of life of *P*. *aeruginosa* by driving the release or the sequestration of one or the other of these two sigma factors.

## Introduction

The study of biofilm biogenesis, composition, and behaviour is of crucial importance in many fields of general interest. Indeed, biofilms are involved in various processes with positive or negative effects. In an environmental context, biofilms can function in clean up processes of soil and water by transforming or degrading toxic compounds as described for bioremediation of Cr(VI) or Ars(III)^1,2^. However, sessile communities are also responsible for microbial-induced corrosion and fouling, presenting economic impacts for industry and in the last case, biofilm formation has to be hampered^3^. When biotic surfaces support biofilms, the association can be either beneficial, as it is for plants that can be protected against soil-borne pathogens or deleterious as it is for humans during chronic infections^4,5^. The impact of biofilms is global; i.e., it is important to understand the various regulatory routes that lead to their biogenesis and dispersion. In *Escherichia coli*, biofilm formation depends on various regulators and among them RpoS. It is the general stress response sigma factor, which allows bacterial survival and adaptation under stressful conditions^6^. In *Pseudomonas aeruginosa* PAO1, the dependence of biofilm formation on RpoS is not as clear^7^. Biofilms formed in flow cell chambers are thicker in the absence of RpoS whereas under chlorine stress, the increase in RpoS is correlated to an increase in biofilm amount^8,9^.

**Figure 1:**
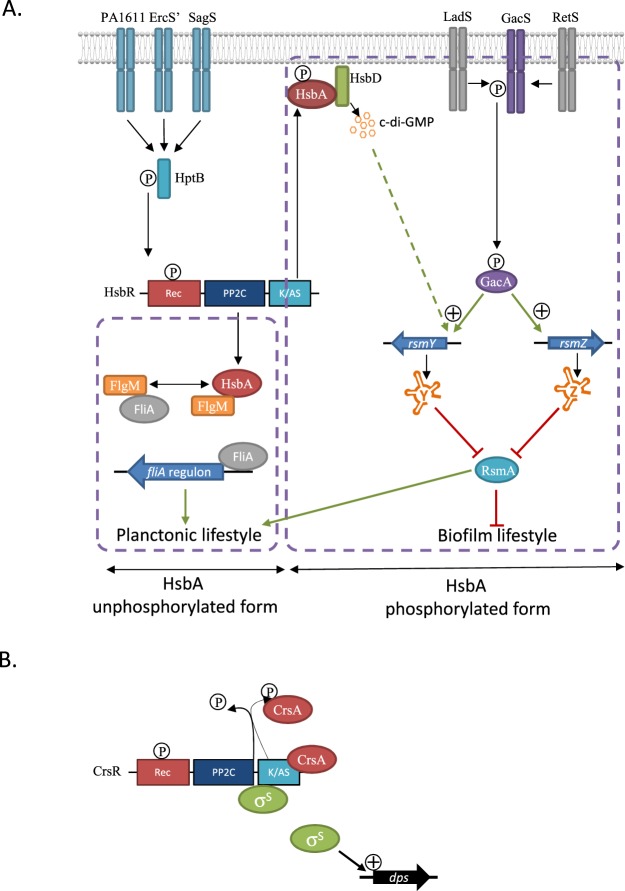
Model for the HtpB/HsbRAD, GacS/GacA/Rsm and CrsRA/RpoS signaling network. (**A**) Unphosphorylated form of HsbR triggers HsbA phosphorylation through its kinase/Anti-σ factor domain (A/KS). Once phosphorylated HsbA binds HsbD diguanylate cyclase and triggers c-di-GMP production which is *rsmY* inducer^27^. RsmY production induces biofilm formation by sequestering/inhibiting RsmA. Phosphorylated form of HsbR triggers HsbA dephosphorylation through its phosphatase domain (PP2C). Once dephosphorylated HsbA binds FlgM anti-σ factor and trigger swimming motility thought FliA σ-factor^25^. HsbA dephosphorylation shutdown biofilm formation by inhibiting HsbD c-di-GMP production. Dashed lines suggest a probable indirect regulation. (**B**) Phosphorylation of CsrA by the CsrR kinase/Anti-σ factor domain (A/KS) leads to dissociation of the CsrA-CsrR complex and a subsequent stable sequestration of RpoS sigma factor by CsrR. Dephosphorylation of CsrA by the CsrR phosphatase domain (PP2C) leads to formation of the CsrA-CsrR complex and a release of the RpoS sigma factor.

*P*. *aeruginosa* is an opportunistic pathogenic bacterium that adopts a sessile lifestyle during chronic infections or a planktonic lifestyle for acute infections. Multiple components have been reported in the literature to regulate motility or biofilm formation in *Pseudomonas* species, including transcriptional regulators, sigma/anti-sigma factors^10–12^, and small RNAs^13^. However, the signals perceived by these different pathways and the interconnections and overlaps between these layers of regulation are still unclear. One of the central two-component signal-transduction systems (TCSs) that is critical to the switch between bacterial sessile and motile lifestyle is the TCS GacS/GacA^14,15^ (Fig. 1A). GacS is an unorthodox histidine kinase (HK) and GacA is its associated response regulator (RR) that functions as a transcriptional regulator which directly and positively controls expression of RsmY and RsmZ, two small non-coding RNAs^16^. RsmY and RsmZ are proposed to be key players in controlling the switch between planktonic and biofilm lifestyles and high expression of the *rsm* gene leads to massive biofilm formation. These two sRNAs out-titer the RNA-binding RsmA translational repressor and release RsmA from its mRNA targets. Titration of RsmA leads to production of biofilm determinants and repression of genes associated with planktonic lifestyle-like flagellar genes^17,18^. The action of the GacS/GacA TCS on these two bacterial small RNAs (sRNAs) is fine-tuned by the sophisticated regulatory network of the TCS, including the RetS, LadS, and Hybrid HK and the HptB pathway^19,20^ (Fig. 1A). The HptB pathway is a multikinase-network in which the three Hybrid HKs PA1611, SagS, and ErcS’ collaborate to detect and integrate signals which lead to the transphosphorylation of the HsbR RR through the HptB protein^21–24^. HsbR contains a receiver domain (Rec), which is the target of the HptB protein, a phosphatase (PP2C) domain, and kinase/anti-sigma factor domain (Figs 1 and S1). HsbR and HsbA, the anti-sigma factor antagonist, form a partner switching system in which HsbR controls the HsbA phosphorylation state through its enzymatic domains^19,25^. A partner switching system implies several partners (e.g., anti-sigma factor, anti-sigma factor antagonist, and sigma factor), which constitute an interaction network that is dependent on the phosphorylation state of the anti-sigma factor antagonist. Thus, the phosphorylation state of HsbA triggers switches between biofilm formation and planktonic lifestyle. Briefly, activation of the HsbR phosphatase domain de-phosphorylates HsbA, and in the presence of this form, HsbA binds the anti-sigma factor FlgM. Consequently, FlgM releases the sigma factor FliA (also named sigma factor^26^), which in return induces flagellar gene expression and promotes the planktonic lifestyle^21,25^. By contrast, when the HsbA protein is phosphorylated by the HsbR kinase domain, HsbA binds to the HsbD diguanylate cyclase, promoting its localisation at cell poles and activating biofilm formation by induction of the *rsmY* gene^27^ (Fig. 1A).

Recently, a novel partner switching system, CrsR-CrsA, was been discovered in the aquatic bacterium *Shewanella oneidensis*^28^. The CrsR-CrsA system is responsible for the post-translational regulation of RpoS (also called sigma S factor) and the specific interaction between CrsR and RpoS was clearly established *in vitro* and *in vivo*^26,29^ (Fig. 1B). CrsR contains the anti-sigma factor domain that, during the exponential phase of bacterial growth, sequesters RpoS and phosphorylates CrsA, the anti-sigma factor antagonist. When cells reach stationary phase, a signal arises and the phosphatase domain of CrsR dephosphorylates CrsA, which in turn binds the anti-sigma factor domain of CrsR. Under these conditions, RpoS is released and can bind the RNA polymerase to express its regulon, inducing the general stress response (Figs 1B and S1). According to bioinformatics analysis, CrsR-CrsA homologs are widespread in the phylum of proteobacteria, among which the HsbR-HsbA system of *P*. *aeruginosa* is included^28,29^.

The aim of this study was to analyse whether in *P*. *aeruginosa*, the HsbR-HsbA partner switch is involved in the post-translational regulation of RpoS, as is the case for the CrsR-CrsA partner switch present in *S*. *oneidensis*. Moreover, the role of RpoS in the switch between sessile and planktonic lifestyles of *P*. *aeruginosa* is questioned.

## Results

### HsbR and CrsR interact with RpoS from *S*. *oneidensis* and *P*. *aeruginosa*

**Figure 2:**
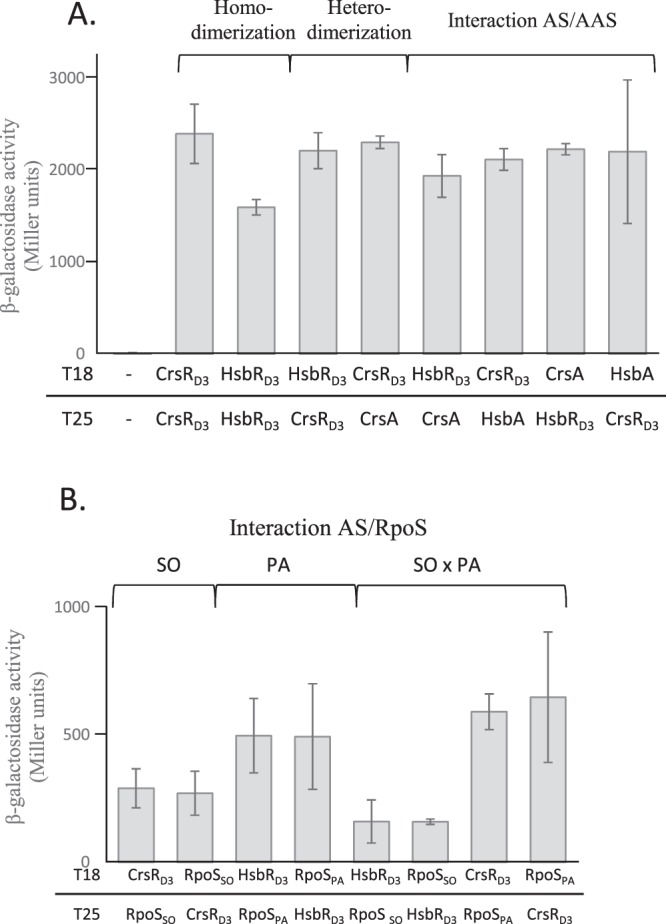
*In vivo* interaction by a two-hybrid assay between the partner-switch proteins of *P. aeruginosa* and *S. oneidensis*. (**A**) Homo-, hetero-dimerization and interaction between the anti-sigma factor domains of CrsR and HsbR and the anti-sigma factor antagonists CrsA and HsbA. (**B**) Interaction between the anti-sigma factor domains of CrsR and HsbR and RpoS from *P. aeruginosa* and *S. oneidensis*. On the left, T18 and T25 indicate the adenylate cyclase domain fused to CrsRD3, HsbRD3, CrsA, HsbA, RpoS from *P. aeruginosa* and *S. oneidensis*, RpoS_PA_ and RpoS_SO_, respectively. The T18 and T25 fusion plasmids were introduced in the *E. coli* BTH101 *clpXP::cat rpoS::tet* strain to prevent degradation of the RpoS protein in *E. coli* and avoid interference caused by RpoS of *E. coli*. Beta-Galactosidase activities are expressed in Miller units. The data from three replicates are presented as means S.D.

HsbR exhibited 25.5% identity and 48.5% similarity with CrsR and shared the same domain organisation; i.e., receiver, phosphatase, and kinase/anti-sigma factor (GHKL ATPase/kinase superfamily) domains (Fig. S1). Moreover, HsbA, the anti-sigma factor antagonist that is phosphorylated by HsbR, possessed 31% and 43% identity and similarity, respectively, with CrsA, the anti-sigma factor antagonist involved with CrsR (Fig. S1). Thus far, the third domain of HsbR is considered for its kinase activity towards HsbA and its ability to bind phosphorylated HsbA but with no sigma factor as a direct target. HsbA is described to be part of a partner-switch involving the anti-sigma factor FlgM, the sigma factor FliA (sigma factor^26^), and the kinase HsbR. Because of the homologies between CrsR and HsbR, and CrsA and HsbA, we hypothesised that HsbR and HsbA constitute a partner-switching system that regulates RpoS in *P*. *aeruginosa*. By bacterial two hybrid experiments, we observed that the anti-sigma factor domains (D3 domains) of CrsR and HsbR were able to form homodimers, as shown in previous studies^20,26^ and more interestingly, they can also interact with one another (Figs 2A and S2 [controls]). This could indicate that the two D3 domains are closely related in structure and function and may play a similar role. Furthermore, we observed that each D3 domain could interact with both anti-sigma factor antagonists (Fig. 2A). It was previously established that CrsRD3 interacts specifically with RpoS in *S*. *oneidensis*^26^. Thus, we tested whether HsbRD3 was also able to bind RpoS of *S*. *oneidensis* (RpoS_SO_). It appears that this last recognised the anti-sigma factor domain D3 of CrsR as expected but also, at a lower level, that of HsbR (Figs 2B and S2 [controls]). This result indicates that the two systems could be functional homologs. To investigate this, we tested, using the same approach, if RpoS from *P*. *aeruginosa* could interact with the D3 domain of HsbR and CrsR. Clearly, HsbRD3 interacted with RpoS from *P*. *aeruginosa* as well as with CrsR. This is an important finding since it indicates that RpoS could be the target of the HsbR-HsbA partner-switch and if true, HsbR-HsbA post-translationally regulates RpoS and therefore the general stress response in *P*. *aeruginosa*. More importantly, since HsbR and HsbA are involved in biofilm biogenesis through sRNA regulation, the connection between RpoS and the HsbR-HsbA partner-switch suggests that RpoS is involved in biofilm biogenesis in this organism.

### RpoS is involved in the regulation of *rsmY* expression

**Figure 3:**
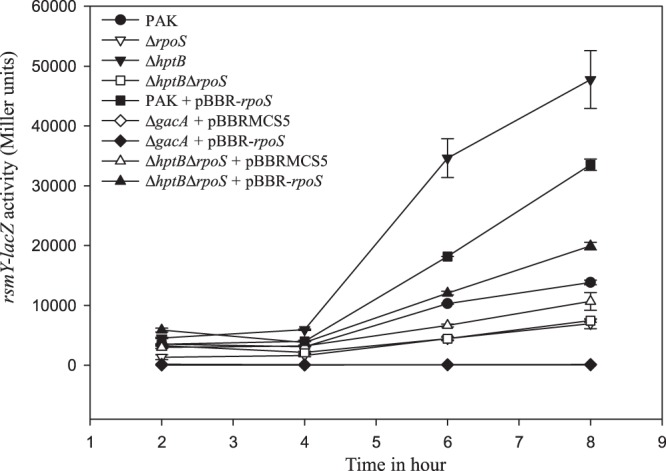
Expression of P*rsmY–lacZ* transcriptional fusion in various *P. aeruginosa* strains. Activity of the transcriptional chromosomal fusions was monitored at different growth stages in various PAK derivated strains overexpressing or not *rpoS* gene. The growth curves of the tested strains were similar (Fig. S4). Corresponding β-galactosidase activities are expressed in Miller units and correspond to mean values (with error bars) obtained from three independent experiments. Statistics performed on values obtained at 6 and 8 hours are presented in Fig. S3.

Biofilm genesis in *P*. *aeruginosa* depends on the presence of sRNA, RsmY, and RsmZ. Expression of the genes *rsmY* and *rsmZ* is positively controlled by the transcriptional regulator GacA; the absence of GacA absence leads to the loss of RsmY and RsmZ production^13,19^. In addition, the expression of *rsmY* is under the control of HsbR, HsbA, and HptB, while that of *rsmZ* is independent^19^.We have shown previously that the biofilm induced by the HptB pathway is abolished in an *rsmY* mutant. Thus, *rsmY* gene activation is an excellent hallmark of biofilm formation triggered by the HptB pathway^19^. To investigate the potential connection between RpoS and the level of RsmY, we engineered an *rpoS* deletion in *P*. *aeruginosa* PAK strain and a plasmid allowing the overproduction of RpoS. First, using a transcriptional fusion between the promoter of *rsmY* (*PrsmY*) and the *lacZ* gene, we assessed P*rsmY* activity in various genetic backgrounds by measuring beta-galactosidase activity. Interestingly, compared to that obtained in the wild-type strain, expression of the P*rsmY*::*lacZ* fusion was significantly decreased in the *rpoS* mutant and increased in the *hptB* mutant (Figs 3 and S3). Moreover, the deletion of *hptB* in the *rpoS* strain showed the same pattern of expression of the reporter fusion as the *rpoS* strain, indicating that *rpoS* is epistatic to *hptB*. By contrast, the induction of a plasmid expressing *rpoS*, introduced in the *hptBrpoS* double mutant, resulted in increased *PrsmY* activity (Figs 3 and S3). Testing the effect of the overproduction of RpoS in the PAK strain, we observed a similar phenotype as P*rsmY::lacZ* fusion expression was almost twice that of the wild-type strain. Altogether, these results indicate that RpoS acts downstream of HtpB in the HtpB cascade and that RpoS is the target of the HsbR/HsbA partner-switch. Finally, when the P*rsmY::lacZ* fusion was introduced in a *gacA*-deleted mutant, no expression was detected and the overexpression of *rpoS* in this context did not change the expression level of the fusion (Fig. 3). This last point indicates that RpoS and GacA act independently on *rsmY* expression and that GacA control is stronger. As a control, we verified that the growth of the tested strains was similar and thus they had no effect on the activity level of the fusion (Fig. S4).

Finally, we confirmed that the regulation of *rsmY* depends on RpoS and not on FliA, the other sigma factor post-translationally regulated by the HsbR-HsbA partner switch^25^, since, as already shown, the absence of FliA had no effect on fusion expression (Fig. S5).

### RpoS promotes biofilm formation

**Figure 4:**
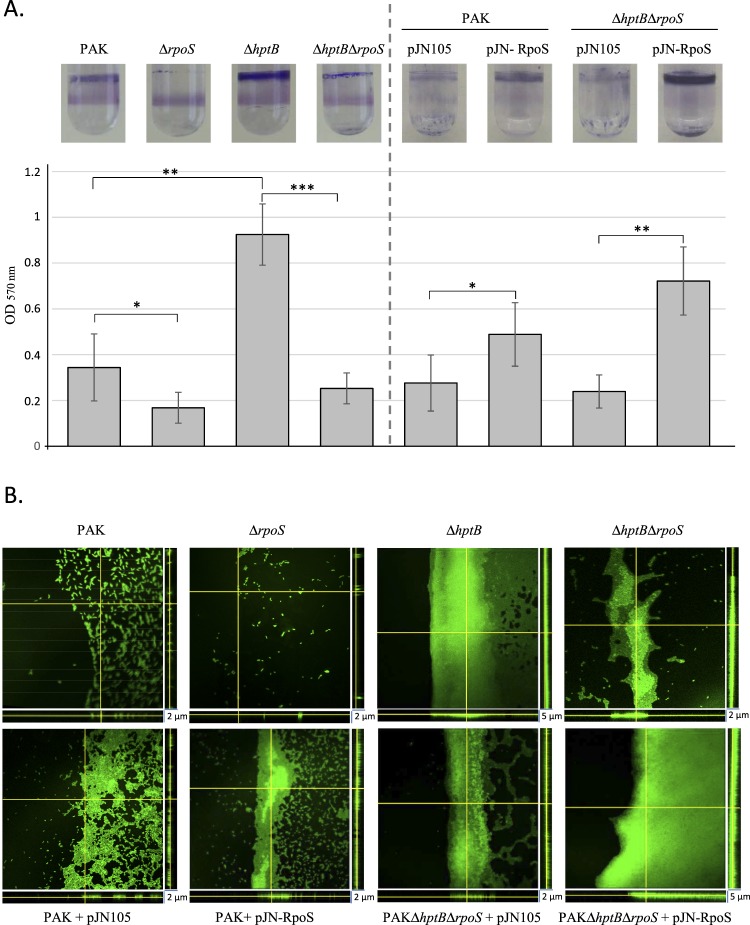
Biofilm formation in various *P. aeruginosa* strains. (**A**) Biofilm production in glass tubes was illustrated (upper panel) and quantified after Crystal Violet-staining (lower panel). Corresponding levels of biofilm production represent mean values and standard deviations obtained from six independent experiments. Wilcoxon-Mann-Whitney tests were performed and *, **, *** and ns referred to p < 0.05, p < 0.01 and p < 0.001 and nonsignificant difference, respectively. (**B**) Biofilm formation was monitored by confocal laser scanning microscopy after 12 h. The extracted z images and their respective xy and xz planes are shown.

These first results indicate that RpoS could have a positive effect on biofilm formation in *P*. *aeruginosa* through the activation of *rsmY* expression. This point was confirmed by the study of an *rpoS* mutant that showed a decrease of 43% in biofilm thickness compared to that obtained with the PAK wild-type strain, shown with glass tube tests and confocal microscopy (Fig. 4). The *rpoS* mutant of *P*. *aeruginosa* PAO1 has been described to exhibit significantly increased biofilm formation in flow cell experiments^8^. To clarify this point, we verified that there was no lag time in biofilm biogenesis in the *rpoS*-deleted strain compared to the wild-type strain that could have hidden a potential hyperbiofilm phenotype (Fig. S6). We propose that the discrepancy of phenotypes reported for the *rpoS* mutant in the literature compared to this study can be due to genetic differences between the strains or more probably to variability in the tested conditions as it has already been shown for *Escherichia coli*^30^. In addition, complementation of the *ΔrpoS* mutant by a plasmid harbouring the *rpoS* gene (pJN-RpoS) restored biofilm biogenesis indicating a direct correlation between RpoS and biofilm formation. Furthermore, strong biofilm synthesis appeared in the wild-type strain only when it harboured and expressed the plasmid pJN-RpoS (Fig. 4A).

To corroborate the results obtained with the P*rsmY*::*lacZ* fusion (Fig. 3), the ability of the *ΔhptBΔrpoS* strain to synthesise a biofilm was tested in glass tubes. The hyperbiofilm phenotype of the Δ*hptB* mutant was abolished in the strain carrying the double deletion since the biofilm detected in the glass tube was 70% thinner than that of the *ΔhptB* strain (Fig. 4A). Moreover, the production of RpoS from pJN-RpoS in the *ΔhptBΔrpoS*strain led to the resaturation of the hyperbiofilm phenotype (Fig. 4A). To further determine the morphology of the biofilm, confocal laser scanning microscopy using 4′,6′-diamidino-2-phenylindole (DAPI)-labelled cells was performed (Fig. 4B). The expression of *rpoS* in the PAK strain led to a modification of the morphology of the biofilm; although there were still isolated cells and it was thin, the biofilm was more compact and homogenous compared to that of the PAK strain. In agreement with the above results, the thick biofilm of the *hptB* strain was significantly decreased when *rpoS* was deleted in this strain (Fig. 4B). Interestingly, in the *ΔhptBΔrpoS* mutant, the overproduction of RpoS led to increased biofilm formation that was thicker than that of the PAK/pJN-RpoS strain. Altogether, these results confirm the positive role of RpoS in the regulation of biofilm synthesis, the presence of RpoS in the HptB pathway, and the hierarchy in the regulation cascade.

**Figure 5:**
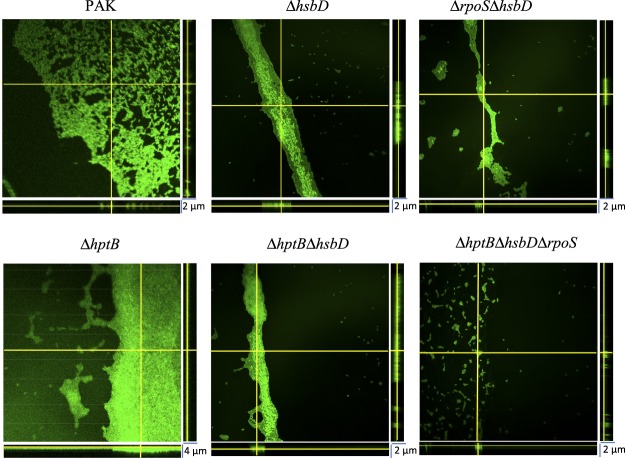
Impact of *hsbD* on biofilm formation in various *P. aeruginosa* strains. Biofilm formation is monitored by confocal laser scanning microscopy after 12 h. The extracted z images and their respective xy and xz planes are shown.

Although the mechanism is not understood, Valentini and co-workers have shown a connection between the HsbD diguanylate cyclase and the HptB pathway during activation of *rsmY* expression^27^ (Fig. 1A). To question a putative link between HsbD and RpoS, we tested biofilm formation of *hsbD* and *rpoS* single and double deletion mutants in a wild type and *hptB* context. As expected, the deletion of only one of these genes led to a decrease in biofilm formation (Fig. 5)^27^, while the *hsbD rpoS* double deletion almost abolished biofilm formation in both the PAK and *hptB* strains, indicating a cumulative effect of the mutations.

### Phosphorylation of HsbA controls the switch in *P*. *aeruginosa* lifestyle

**Figure 6:**
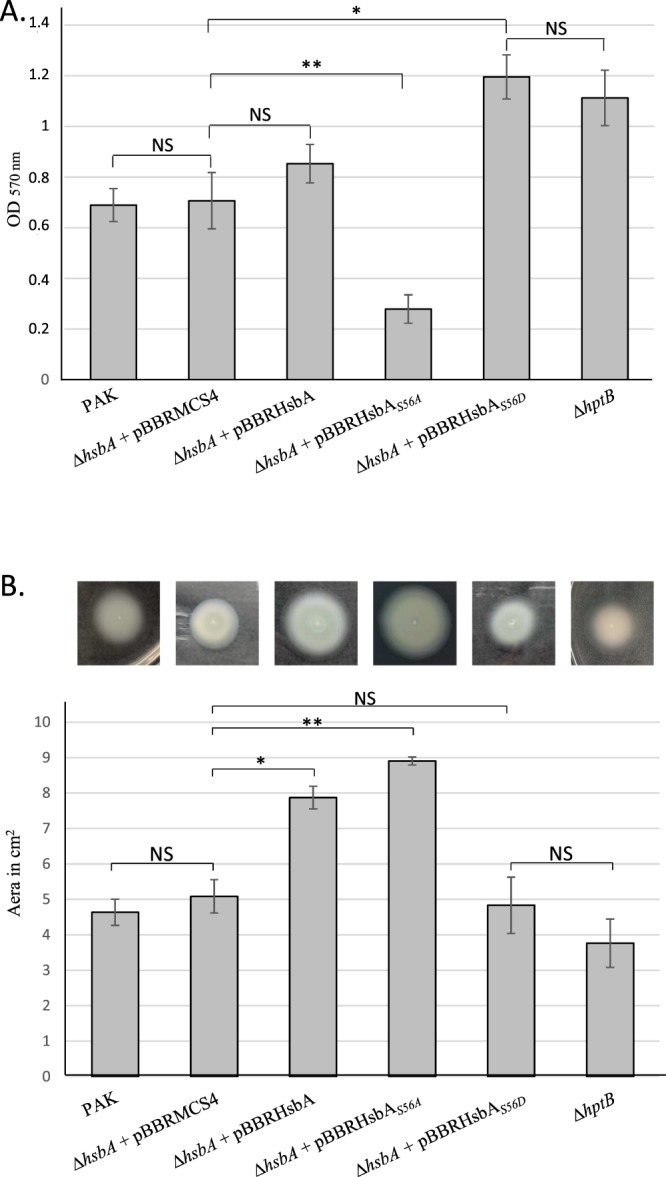
Impact of HsbA variant on biofilm formation and swimming motility. (**A**) Quantification of biofilm production after Crystal Violet-staining. Corresponding levels of biofilm production represent mean values and standard deviations obtained from three independent experiments. (**B**) Swimming mobility on soft agar plate was illustrated (upper panel) and colony size surface was measured (lower panel). Colony surface is expressed in square cm and correspond to mean values and standard deviations obtained from three independent experiments. Wilcoxon-Mann-Whitney tests were performed and *, **, *** and ns referred to p < 0.05, p < 0.01 and p < 0.001 and nonsignificant difference, respectively.

The interaction between HsbR and HsbA occurs when HsbA is phosphorylated on Ser_56_^25,27^. Under these conditions, we hypothesised that RpoS is not bound to HsbR and thus can positively control genes of its regulon, such as *rsmY*, leading to biofilm formation. Otherwise, when not phosphorylated on Ser_56_, HsbA binds FlgM. The sigma factor FliA is freed and available to positively regulate the swimming process. It is likely, that according to environmental cues, *P*. *aeruginosa* could choose between biofilm formation and swimming and the state of the bacterium depends on the phosphorylation status of HsbA. To mimic and control the phosphorylation state of HsbA, we engineered a strain deleted for *hsbA* and harbouring a plasmid allowing the production of native HsbA or two HsbA variants, HsbA_S56A_ and HsbA_S56D_, mimicking its non-phosphorylated or phosphorylated forms, respectively. Finally, recombinant *hsbA* strains were tested for their ability to synthesise a biofilm and to swim. In the absence of HsbA, bacteria formed a biofilm and swam at a level that was independent of the HptB pathway, which was considered the reference for the experiment (Fig. 6A,B). The production of wild-type HsbA in the mutant drove the cells toward a planktonic state since an increase of 40% of swimming was observed compared to the mutant. Complementation of the deleted strain by HsbA_S56A_ led to an 80% increase in the swimming capacity of the recombinant strain whereas its ability to form a biofilm was decreased by 60%. This indicates that the unphosphorylated state of HsbA mimicked here by the Ser56Ala substitution favours the planktonic state of the bacteria, which corresponds to a release of FliA and a sequestration of RpoS (Fig. 6). Under these conditions, genes involved in swimming are activated while those involved in biofilm formation and under RpoS regulation are repressed. By contrast, when the deleted strain was complemented with HsbA_S56D_, biofilm formation was increased by 45% compared to the deleted strain while swimming was similar to that of the *hsbA* strain (Fig. 6). These results indicate that when the substitution mimics the phosphorylated state of HsbA, the bacteria are rather organised in biofilm structure rather than planktonic and swimming. Under these conditions, RpoS is released from HsbR and FliA is sequestered by FlgM (Fig. 6). Thus, it appears that the balance between phosphorylated and dephosphorylated HsbA drives biofilm formation or the swimming ability of the bacterium.

## Discussion

**Figure 7:**
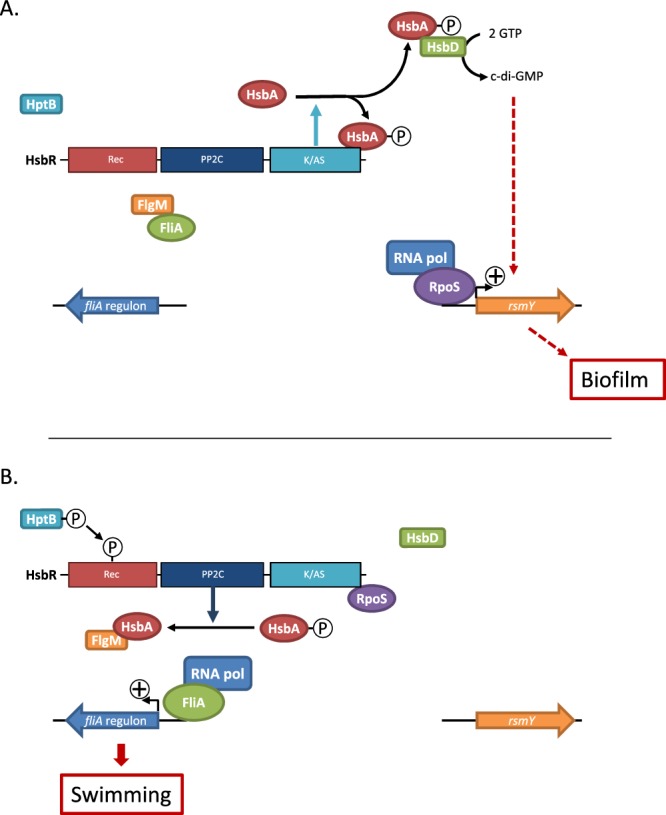
The HtpB/HsbRAD/RpoS signaling network. (**A**) Unphosphorylated form of HsbR triggers HsbA phosphorylation through its kinase/Anti-σ domain (D3 domain). Once phosphorylated HsbA binds HsbR kinase/Anti-σ factor domain and HsbD diguanylate cyclase, this binding trigger both the releases of RpoS σ-factor and c-di-GMP production which are biofilm-inducing. (**B**) Phosphorylated form of HsbR triggers HsbA dephosphorylation through its phosphatase domain. Once dephosphorylated HsbA binds FlgM anti-σ factor and release HsbR kinase/Anti-σ factor domain and HsbD. Under its none-phosphorylated form HsbA binds FlgM and triggers swimming motility thought FliA σ-factor. HsbA dephosphorylation shuts down biofilm formation by inhibiting HsbD c-di-GMP production and by sequestration of RpoS by HsbR.

In *P*. *aeruginosa*, approximately 700 genes are under the direct or indirect control of RpoS, and most are related to virulence and quorum sensing^31^. Among them, a positive regulation of the *pls* gene involved in the production of biofilm matrix polysaccharides production has been reported^10^. Therefore, it is of high importance to understand the different levels of RpoS regulation. To our knowledge, little is known about the post-translational regulation of RpoS in *P*. *aeruginosa* compared to that of *E*. *coli*, for example^32,33^. However, it has been shown that RpoS is degraded by ClpXP machinery during exponential growth^34^. In *E*. *coli*, post-translational regulation of RpoS depends on RssB, which binds the sigma factor to deliver it to the ClpXP machinery. In the presence of stress, Ira proteins are produced (IraP, IraM, IraD) and bind RssB to free RpoS, which binds RNA polymerase and activates or represses genes of its regulon^35^. In this study, we unveiled that in *P*. *aeruginosa*, RpoS is controlled at the post-translational level by the HsbR-HsbA partner switch. In *S*. *oneidensis*, CrsR sequesters RpoS during the exponential phase of growth and releases it when a signal arises or when the bacterium enters stationary phase. It is likely that in *P*. *aeruginosa* a similar mechanism controls the release and sequestration of RpoS, preventing its complete degradation by the Clp machinery and keeping it rapidly available. It is noteworthy that the mechanism of action of the two systems, CrsR-CrsA and HsbR-HsbA, present differences since CrsR binds CrsA when the latter is not phosphorylated whereas HsbR binds phosphorylated HsbA (Fig. 7)^21,26,27^. Moreover, phosphorylated HsbA recognises two partners since it has recently been shown to also bind HsbD, a diguanylate cyclase^36^. The binding between HsbD and phosphorylated HsbA supports biofilm biogenesis and drives localisation of the complex to the cell pole^27^. It would be interesting in further investigations to determine whether a complex with the three proteins exists at the pole of the cell. *hsbR* and *hsbA* expression is positively regulated by RpoS^31^, suggesting that the production of HsbR and HsbA is enhanced when RpoS is required during stationary phase or for biofilm biogenesis. Finally, the discovery that in *P*. *aeruginosa*, the HsbR-HsbA partner-switch regulates the sigma factor of the general stress response corroborates the notion that CrsR-CrsA homologous systems interplay in a general manner in the post-translational regulation of RpoS in various bacteria.

The second major point of this work is that RpoS controls biofilm formation. It acts at least through the regulation of RsmY by the HptB pathway. The control of biofilm formation by RpoS through the activation of sRNAs has been reported in other bacteria. For instance, in *E*. *coli* and *Salmonella*, three sRNA (SdsR, GadY, and SraL) genes are part of the RpoS regulon. Expression of these non-coding RNAs triggers biofilm formation in these two organisms^6^. In *P*. *aeruginosa*, under our conditions, the absence of RpoS led to a decrease in biofilm thickness and the presence of more planktonic cells while its overproduction reinforced biofilm homogeneity and thickness. The epistasis experiments established that the control of RpoS by HsbR-HsbA was part of the HptB pathway and independent of the GacA pathway. We also showed that although HsbD intersects with the HptB pathway to control biofilm formation^27^, activation by RpoS is independent of that of HsbD.

Furthermore, we demonstrated that RpoS regulation by HsbR-HsbA is connected to regulation of the FliA sigma factor under the control of the FlgM-HsbA partner-switch. The two partner-switching systems form a network controlling the availability of two sigma factors that in turn control two cellular processes with antagonist effects. While RpoS is necessary for a community way of life, FliA drives the swimming process. The common element of these two systems is the anti-sigma factor antagonist HsbA, which is responsible for the switch between these two lifestyles. It is noteworthy that complementation of the *hsbA* mutant by a plasmid producing HsbA seems to favour a planktonic lifestyle, likely because under the tested experimental conditions, HsbA was produced to a greater extent than HsbR. As shown by mimicking the phosphorylated or non-phosphorylated state of HsbA, the bacterium chooses to form a biofilm or to swim, respectively. The signal transduced by the HptB pathway leads to activation of the phosphatase borne by the second domain of HsbR and thus to the dephosphrylation of HsbA, which can in turn bind FlgM and liberate FliA. Therefore, we suggest that the interconnection of the two systems drives biofilm formation and likely also its dispersal. According to environmental cues and the generated benefits, bacteria will trigger one or the other of these two modes of life. A recent study in *Vibrio cholerae* indicated that the signal input of biofilm dispersal combines both the individual stress response and collective information related by quorum sensing^37^. It is likely that this is also the case in *P*. *aeruginosa*^38^. This is reminiscent of the conclusions of a recent study^27^, which indicated that the adenylate cyclase HsbD is involved in biofilm biogenesis as well as in the various motilities of bacteria. These conclusions are in perfect agreement with our results since HsbD binds phosphorylated HsbA, and the phosphorylation state of HsbA triggers one of the two lifestyles. Moreover, it highlights once again the complexity of the process driving the switch between the various life states offered to the bacterium. The choice made appears like a jigsaw in which each piece moves the balance to one or the other side.

In conclusion, we have shown that the post-translational regulation of RpoS in *P*. *aeruginosa* depends on the HsbR-HsbA partner-switch. This system is connected to the FlgM-HsbA partner-switch and HsbA, according to its phosphorylation state, triggers the release or sequestration of RpoS and FliA. The connection of these two mechanisms with antagonist goals is likely the most efficient system the bacteria has found to decide to swim or to form and disperse a biofilm.

## Materials and Methods

### Bacterial strains, growth conditions, and media

The bacterial strains and plasmids used in this study are described in Table S1 and the oligonucleotides used in Table S2. Strains were grown aerobically in Luria–Bertani (LB) broth or on LB agar at 37 °C or 30 °C. Plasmids were introduced into *P*. *aeruginosa* by triparental mating using the conjugative properties of pRK2013. The transformants were selected on *Pseudomonas* isolation agar. Antibiotics were used at the following concentrations for *E*. *coli*: 50 µg/mL ampicillin, 50 µg/mL streptomycin, 25 µg/mL kanamycin, and 15 µg/mL tetracycline. For *P*. *aeruginosa*, 500 µg/mL carbenicillin, 2000 µg/mL streptomycin, 60 µg/mL gentamicin, and 200 µg/mL tetracycline were used.

### Bacterial two-hybrid experiments

The DNA regions encoding *rpoS* were PCR amplified using PAK or *Shewanella oneidensis* MR-1 genomic DNA as templates with appropriate oligonucleotide pairs (Table S2). PCR products were digested by *XbaI*/*KpnI* of PAK RpoS or *EcoRI/XhoI* of SO RpoS and cloned into pKT25 or pUT18C, yielding pKT25-*rpoS* and pUT18C-*rpoS*, respectively. The adenylate cyclase-deficient *E*. *coli* strain BA159 (BTH101 *clpXP::cat rpoS::tet*) was used to screen for positive interactions^39^. BTH101 competent cells were transformed simultaneously with pKT25 and pUT18C derivatives and transformants were selected on agar plates supplemented with ampicillin (100 μg/mL) and kanamycin (50 μg/mL) after 2 days of growth at 30 °C. Cell treatment and measurement of beta-galactosidase activity were performed as previously described^13^.

### Measurement of β-galactosidase activity

Strains carrying the *lacZ* transcriptional fusions were grown in LB under agitation at 37 °C in the presence of 0.5% arabinose to induce pJN105 promoter expression. Bacterial cells were collected by centrifugation at different growth times. β-galactosidase activity was measured using the method developed by Miller.

### Biofilm assay

*P. aeruginosa* adherence assays were performed in individual glass tubes containing 1 mL of medium as described previously^40^. Bacteria were grown in M63 medium supplemented with 1 mM MgCl_2_, 0.5% casa-amino acids, and 0.2% glucose under static conditions at 30 °C. After 12 h, the cultures were incubated with 1% Crystal Violet for 10 min to stain the attached bacteria and washed twice. Staining was extracted by treatment with 400 μL of 95% ethanol and 600 μL of water. All quantification assays measured at 570 nm were performed at least in triplicate. For confocal laser scanning microscopy analysis of biofilms, *P*. *aeruginosa* strains were grown in a 4-well chambered coverglass (Lab-teK II). M63 derivate medium (200 μL) containing a bacterial suspension at OD_600_ of 0.1 was incubated at 30 °C. After 12 h, the medium was removed from each chamber and rinsed twice by adding 200 μL of sterile phosphate-buffered saline to remove unattached cells. Prior to observation, bacteria were fixed with 4% paraformaldehyde and stained using DAPI for 15 min. Images were taken at locations of biofilm formation using a confocal laser scanning microscope Confocal Olympus FV1000. Positions were chosen for sagittal sections (xz position) to minimise experimental variability. The number of images for three-dimensional biofilm observation within each stack depended on biofilm thickness. All confocal images were analysed using ImageJ software^41^.

### Construction of deletion mutants

PCR was used to generate a 500 bp DNA fragment upstream (Up) and a 500 bp DNA fragment downstream (Dn) of the *hsbD*, *rpoS* and *fliA* genes using the appropriate pairs of primers (Table S2). Upstream and downstream PCR products were linked together by overlapping PCR and products were digested with *Xba*I and *Spe*I and cloned in the suicide vector pKNG101, yielding pKNG101Δ*hsbD*, pKNG101Δ*rpoS* and pKNG101Δ*fliA*, respectively. The suicide plasmids were introduced into *P*. *aeruginosa* via a three-partner procedure and the deletion mutants were obtained by double selection on PIA agar plates supplemented with streptomycin (1000 µg/mL) at 37 °C and NaCl-free LB agar containing 6% sucrose at 30 °C. The PAKΔ*hptB*Δ*rpoS* and PAKΔ*hptB*Δ*fliA* double mutants were constructed as follows: the pKNG101Δ*fliA* or pKNG101Δ*rpoS* vector was introduced by mating into the PAKΔ*hptB*. The PAKΔ*hsbD*Δ*rpoS*, PAKΔ*hptB*Δ*hsbD* double mutants and the PAKΔ*hptB*Δ*hsbD*Δ*rpoS* were constructed as follows: the pKNG101Δ*hsbD* vector was introduced by mating into the PAKΔ*hptB*, PAKΔ*rpoS and* PAKΔ*hptB*Δ*rpoS*.

### Gene overexpression

A DNA fragment corresponding to the *rpoS* open reading frame was amplified by PCR using appropriate oligonucleotide pairs (Table S2) and cloned into the pBBRMCS5 vector yielding pBBR-*rpoS*. After DNA sequencing, pBBR-*rpoS* was digested using *EcoRI*/*XbaI* for subcloning into pJN105, yielding pJN-*rpoS*. The *hsbA*_*S56A*_ and *hsbA*_*S56D*_ variants were constructed using the quick exchange site-directed mutagenesis method. Briefly, the serine residue at position 56 was substituted with an alanine or glutamine residue. This was done by using the pBBR-*hsbR* vector as matrice and by PCR using *pfu* turbo DNA polymerase (Stratagene) and 39-mer primers that incorporated appropriate mismatches to introduce the expected mutations (Table S2). The resulting PCR products were digested with *Dpn*I for 1 h and introduced into *E*. *coli* by transformation.

### Swimming assays

Swimming assays were carried out on 10 g/L tryptone, 5 g/L NaCl, and 0.3% agar plates. Standardised overnight culture (0.5 μL) was deposited below the surface of the agar and plates were incubated at 30 °C overnight. Pictures were taken from a representative plate out of four independent experiments. ImageJ software was used to determine the area of the plate surface covered by the bacteria.

### Significance

This work demonstrates that the choice made by *P. aeruginosa* between forming biofilms or swimming results from a connection between two partner-switches sharing the same anti sigma factor antagonist. We established that HsbR is an anti-sigma factor controlling RpoS and unveiled a connection between the HptB-HsbR-HsbA signal transduction cascade and the RpoS pathway during biofilm biogenesis. Another important finding focuses on the role of the anti-sigma factor antagonist HsbA in the balance between sessile and planktonic life styles. Altogether these results increase our knowledge of the mechanism driving the switch between these two ways of life, which are crucial to control *P. aeruginosa* in its various biotopes and clinical contexts.

## Supplementary information

Supplementary material
