# Supplementary material for: Connected partner-switches control the life style of Pseudomonas aeruginosa through RpoS regulation
Source: Sci Rep. 2019 Apr 24;9:6496. doi: 10.1038/s41598-019-42653-5 (PMC6482189; doi:10.1038/s41598-019-42653-5)
Supplement: Supplementary file 1 — Supplementary material [file 41598_2019_42653_MOESM1_ESM.pdf]

Supplementary Information for

Connected partner-switches control the life style of *Pseudomonas aeruginosa* through RpoS regulation

Sophie Bouillet[a], Moly Ba[b], Laetitia Houot[b], Chantal Iobbi-Nivol[a1] and Christophe Bordi[b1]

[a] Aix Marseille Univ, CNRS, BIP, Marseille, France
[b] Aix Marseille Univ, CNRS, LISM, Marseille, France

[1]To whom correspondence should be addressed; e-mail: bordi@imm.cnrs.fr, iobbi@imm.cnrs.fr

This PDF file includes:

Figs. S1 to S6
Tables S1 and S2
Supplementary references

Fig. S1 Domain organization of CrsR and HsbR response regulators.

Schematic representation of the three domains of CrsR and HsbR. CrsR contains 565 amino acids and HsbR 571. The N-terminal regulatory receiver domain (D1, residues 1–118 and 13-125 for CrsR and HsbR, respectively), the central PP2C phosphatase domain (D2, residues 193–391 and 188-336 for CrsR and HsbR, respectively), and the C-terminal GHKL (gyrase, Hsp90, histidine kinase, MutL) ATPase and anti-sigma factor domain (D3, residues 407–565 and 445-563 for CrsR and HsbR, respectively) are shown. The level of identity between homologous domains (in %) is indicated. Domains were defined using Pfam database.

Fig S2. Control of i*n vivo* interaction by a two-hybrid assay.

Each fusion protein in both T18 and T25 vectors were tested against the empty counterpart vector. Positive (T18zip/T25zip) and negative (empty vectors) controls were added. Galactosidase activities are expressed in miller units. The data from three replicates are presented as means S.D.

Fig S3. Statistical analysis of P*rsmY–lacZ* transcriptional fusion in various *P. aeruginosa* strains. Activity was recorded after 6 and 8 hours of growth. Corresponding β-galactosidase activities are expressed in Miller units and correspond to mean values (with error bars) obtained from three independent experiments. Wilcoxon-Mann-Whitney tests were performed, and *, ** and *** referred to $p<0.05$, $p<0.01$ and $p<0.001$, respectively.

Fig S4. Growth curves of the *P. aeruginosa* strains used to study P*rsmY–lacZ* transcriptional expression.

For each strain the OD_{600} nm value correspond to mean values (with error bars) obtained from three independent experiments

Fig. S5 Expression of P*rsmY–lacZ* transcriptional fusion in PAK, PAKΔ*hptB*, PAKΔ*fliA* or PAKΔ*hptBfliA* strains. Activity was recorded after 4h growth. Corresponding β-galactosidase activities are expressed in Miller units and correspond to mean values (with error bars) obtained from three independent experiments. Wilcoxon-Mann-Whitney tests were performed, and ns referred to nonsignificant difference.

Fig. S6 Biofilm formation in PAK and PAKΔ*rpoS* at different growth stages. (A) Biofilm production monitored at different growth stages in glass tubes (upper panel) and quantified after Crystal Violet-staining (lower panel). Corresponding levels of biofilm production represent mean values and standard deviations obtained from six independent experiments. (B) Biofilm formation is monitored by confocal laser scanning microscopy at the time indicated in the figure. The extracted z images and their respective xy and xz planes are shown.

Table S1: Strains and plasmids used in this study

Strains	Relevant characteristics*	Source
E. coli		
DH5α	*endA1 hsdR17 supE44 thi-1 recA1 gyrA relA1 Δ(lacZYA-argF)U169 deoR (phi 80lacZ Δ M15)*	Lab collection
BA159	BTH101 (F- *cya-99 araD139 galE15 galK16 rpsL1* (Sm^r) *hsdR2 mcrA1 mcrB1*) *clpXP::cat rpoS::tet*	(1)
CC118(λpir)	Host strain for pKNG101 replication, Δ(*ara-leu*) *araD* Δ*lac*X74 *galE galK phoA20 thi-1 rpsE rpoB argE*(Am) *recA1* Rf^R (λpir)	Lab collection
P. aeruginosa		
PAK	Wild-type	(2)
PAKΔ*htpB*	PAK deletion mutant for *hptB* gene	(3)
PAKΔ*rpoS*	PAK deletion mutant for *rpoS* gene	This study
PAKΔ*hsbD*	PAK deletion mutant for *hsbD* gene	This study
PAKΔ*htpB*Δ*rpoS*	PAK deletion mutant for *hptB* and *rpoS* genes	This study
PAKΔ*hsbD*Δ*rpoS*	PAK deletion mutant for *hsbD* and *rpoS* genes	This study
PAKΔ*hptB*Δ*hsbD*	PAK deletion mutant for *hsbD* and *htpB* genes	This study
PAKΔ*hptB*Δ*hsbD*Δ*rpoS*	PAK deletion mutant for *hptB*, *hsbD* and *rpoS* genes	This study
PAKΔ*hsbA*	PAK deletion mutant for *hsbA* gene	(3)
PAKΔ*fliA*	PAK deletion mutant for f*liA* gene	This study
PAKΔ*htpB*Δ*fliA*	PAK deletion mutant for *hptB* and *fliA* genes	This study
PAK*attB::rsmY-lacZ*	PAK strain with *rsmY-lacZ* inserted at *attB* sites	(4)
PAKΔ*rpoSattB::rsmY-lacZ*	PAKΔ*rpoS* strain with *rsmY-lacZ* inserted at *attB* sites	This study
PAKΔ*htpBattB::rsmY-lacZ*	PAKΔ*htpB* strain with *rsmY -lacZ* inserted at *attB* sites	This study
PAKΔ*htpB*Δ*rpoSattB::rsmY-lacZ*	PAKΔ*htpB*Δ*rpoS* strain with *rsmY-lacZ* inserted at *attB* sites	This study
PAKΔ*gacAattB:: rsmY-lacZ*	PAKΔ*gacA* strain with *rsmY -lacZ* inserted at *attB* sites	(4)
**Plasmid**	**Relevant characteristics**	**Source**
pUT18C	Two-hybrid plasmid, *cyaAT18* fusion, Ap^R	(5)
pUT18C-*crsRD3*	Two-hybrid plasmid containing *cyaAT18-crsR* D3 domain fusion, Ap^R	(6)
pUT18C-*hsbRD3*	Two-hybrid plasmid containing *cyaAT18- hsbR* D3 domain fusion, Ap^R	(3)
pUT18C- *rpoSso*	Two-hybrid plasmid containing *cyaAT18- rpoS S. oneidensis* fusion, Ap^R	(6)
pUT18C- *rpoSpa*	Two-hybrid plasmid containing *cyaAT18- rpoS P. aeruginosa* domain fusion, Ap^R	This study
pKT25	Two-hybrid plasmid, *cyaAT25* fusion, Km^R	(5)
pKT25-*crsRD3*	Two-hybrid plasmid containing *cyaAT25– crsR* D3 domain fusion, Km^R	(6)
pKT25-*hsbRD3*	Two-hybrid plasmid containing *cyaAT25– hsbR* D3 domain fusion, Km^R	(7)
pKT25-*rpoSso*	Two-hybrid plasmid containing *cyaAT25– rpoS S. oneidensis* fusion, Km^R	This study
pKT25- *rpoSpa*	Two-hybrid plasmid containing *cyaAT25– rpoS P. aeruginosa* fusion, Km^R	This study
miniCTX-*lacZ*	Tc^r *lacZ*$^+$; self-proficient integration vector with *tet*, V-*FRT-attPMCS*, *ori*, *int*, and *oriT*	(8)
miniCTX-*rsmY-lacZ*	Promoter region of *rsmY* gene inserted into miniCTX-*lacZ*, Tc^R	(4)
pBBRMCS5	Broad host range plasmid, Gm^R	(8)
pBBR-*rpoS*	pBBRMCS5 carrying the *rpoS* gene, Gm^R	This study
pBBRMCS4	Broad host range plasmid, Ap^R	(8)
pBBR-*hsbA*	pBBRMCS4 carrying the *hsbA* gene, Ap^R	(3)
pBBR-*hsbA*$_{S56A}$	pBBRMCS4 carrying the *hsbA* gene with *S56A* substitution, Ap^R	This study
pBBR-*hsbA*$_{S56D}$	pBBRMCS4 carrying the *hsbA* gene with *S56D* substitution, Ap^R	This study
pRK2013	Tra$^+$ Mob$^+$ Km^R	Lab collection
pKNG101Δ*rpoS*	Mutator plasmid for *rpoS* deletion Sm^R	This study
pKNG101Δ*fliA*	Mutator plasmid for *fliA* deletion Sm^R	This study
pJN105	araC-pBAD expression vector, Gm^R	Lab collection
pJN-RpoS	pJN105 carrying the *rpoS* gene, Gm^R	This study

* Sm^R , streptomycin resistance, Gm^R gentamicin resistance, Ap^R ampicillin resistance, Km^R kanamycin resistance and Tc^R tetracycline resistance

Table S2: Oligonucleotides used in this study

Names	Oligonucleotides (5’→3’)
**Two-Hybrid**	
DH_PAK UP_RpoS	CCTCTAGAGATGGCACTCAAAAAAGAAGGG
DH_PAK Do_RpoS	GGGGTACCTCACTGGAACAGCGCGTCACTCG
DH SO UP RpoS	TAGAATTCATGAGCCGCATAAATAGCACTG
DH SO DO RpoS	TACTCGAGTTAATTTCTAAATAGAGCCTC
**Gene Deletion**	
UpUFliA	CCGGATCCGCACCTCTCGCCGATGCAGC
UpDFliA	TGGCCGGAGCTGGCACGGCCGAACCTGTCGCGGG
DnUFliA	AGGTTCGGCCGTACCGGCCTCGACCGCAGC
DnDFliA	GGACTAGTCACCGCCTGCTGGAAGTGCC
UpURpoS	CCCCCCCCTGCAGGTCGACGGATCCTACGTCGGTACCTGCCAAGC
UpDRpoS	GGTCTAAGGTTTTCCGTCACCATGTCGTTATCCCTTGCATGAGTTCG
DnURpoS	TGCAAGGGATAACGACATGGTGACGGAAAACCTTAGACCC
DnDRpoS	TTCTACTTATGGTACCCGGGGATCCGAGAAGAAGGATGCCCTG
UpUHsbD	CCCTGCAGGTCGACGGATGCGCTCGCTATCCGACATGG
UpDHsbD	TGCGCCATGGACTCACACCTCTTCTCTTGGAGGGCTTGG
DnUHsbD	CCAAGAGAAGAGGTGTGAGTCCATGGCCGATTTTGTCC
DnDHsbD	CTTATGGTACCCGGGGATCCGGTTCTCCAGGCGTAGCAGG
**Quick change mutations**	
HsbA-S56A-QC-F	ACTTACCTGGACGCGTCGGCCCTCGGC
HsbA-S56A-QC-R	GCCGAGGGCCGACGCGTCCAGGTAAGT
HsbA-S56D-QC-F	ACTTACCTGGACGATTCGGCCCTCGGC
HsbA-S56D-QC-R	GCCGAGGGCCGAATCGTCCAGGTAAGT
**Gene expression**	
Up_RopS_prod	TTCCTGCAGCCCGGGGGATCTCCAGCGGGAAAGGAATCGC
Do_RpoS_prod	GCCGCTCTAGAACTAGTGGATCTGTAAGTTAATGCTTACAAGAGC

Supplementary references

1. Battesti A, Bouveret E (2012) The bacterial two-hybrid system based on adenylate cyclase reconstitution in Escherichia coli. *Methods* 58(4):325–34.
2. Sastry PA, Pearlstone JR, Smillie LB, Paranchych W (1983) Amino acid sequence of pilin isolated from pseudomonas aeruginosa PAK. *FEBS Lett* 151(2):253–6.
3. Bordi C, et al. (2010) Regulatory RNAs and the HptB/RetS signalling pathways fine-tune Pseudomonas aeruginosa pathogenesis. *Mol Microbiol* 76(6):1427–1443.
4. Chambonnier G, et al. (2016) The Hybrid Histidine Kinase LadS Forms a Multicomponent Signal Transduction System with the GacS/GacA Two-Component System in Pseudomonas aeruginosa. *PLOS Genet* 12(5):e1006032.
5. Karimova G, Ullmann A, Ladant D (2000) A bacterial two-hybrid system that exploits a cAMP signaling cascade in Escherichia coli. *Methods Enzymol* 328:59–73.
6. Bouillet S, et al. (2016) The General Stress Response σS Is Regulated by a Partner Switch in the Gram-negative Bacterium Shewanella oneidensis. *J Biol Chem* 291(50):26151–26163.
7. Houot L, Fanni A, de Bentzmann S, Bordi C (2012) A bacterial two-hybrid genome fragment library for deciphering regulatory networks of the opportunistic pathogen Pseudomonas aeruginosa. *Microbiology* 158(Pt_8):1964–1971.
8. Hoang TT, Kutchma AJ, Becher A, Schweizer HP (2000) Integration-proficient plasmids for Pseudomonas aeruginosa: site-specific integration and use for engineering of reporter and expression strains. *Plasmid* 43(1):59–72.

Figure S1

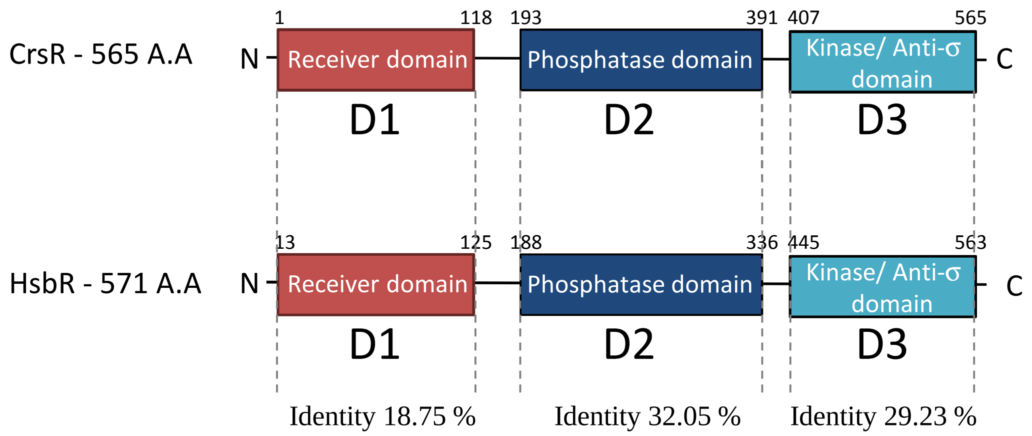

CrsR - 565 A.A
1
118
193
391
407
565
N
Receiver domain
Phosphatase domain
Kinase/ Anti-σ domain
C
D1
D2
D3
HsbR - 571 A.A
13
125
188
336
445
563
N
Receiver domain
Phosphatase domain
Kinase/ Anti-σ domain
C
D1
D2
D3
Identity 18.75 %
Identity 32.05 %
Identity 29.23 %

Figure S2

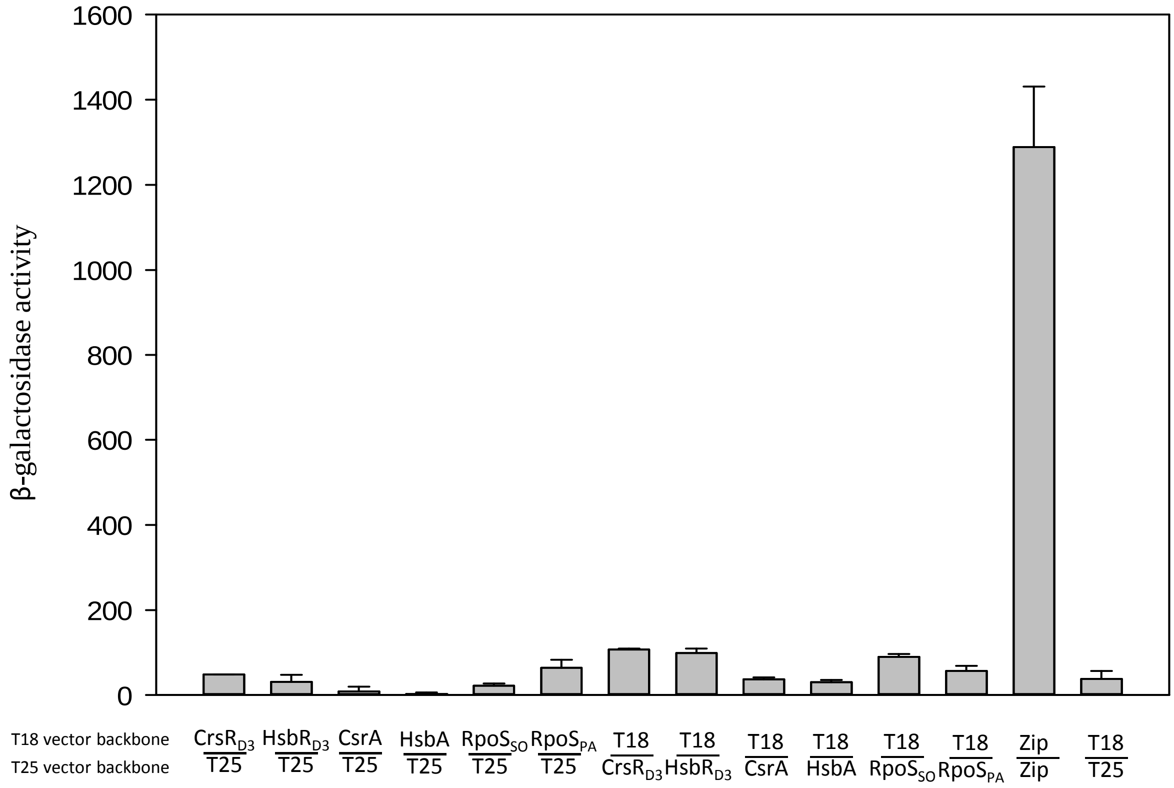

β-galactosidase activity
0
200
400
600
800
1000
1200
1400
1600
T18 vector backbone
T25 vector backbone
CrsR$_{D3}$ / T25
HsbR$_{D3}$ / T25
CsrA / T25
HsbA / T25
RpoS$_{SO}$ / T25
RpoS$_{PA}$ / T25
T18 / CrsR$_{D3}$
T18 / HsbR$_{D3}$
T18 / CsrA
T18 / HsbA
T18 / RpoS$_{SO}$
T18 / RpoS$_{PA}$
Zip / Zip
T18 / T25

Figure S3

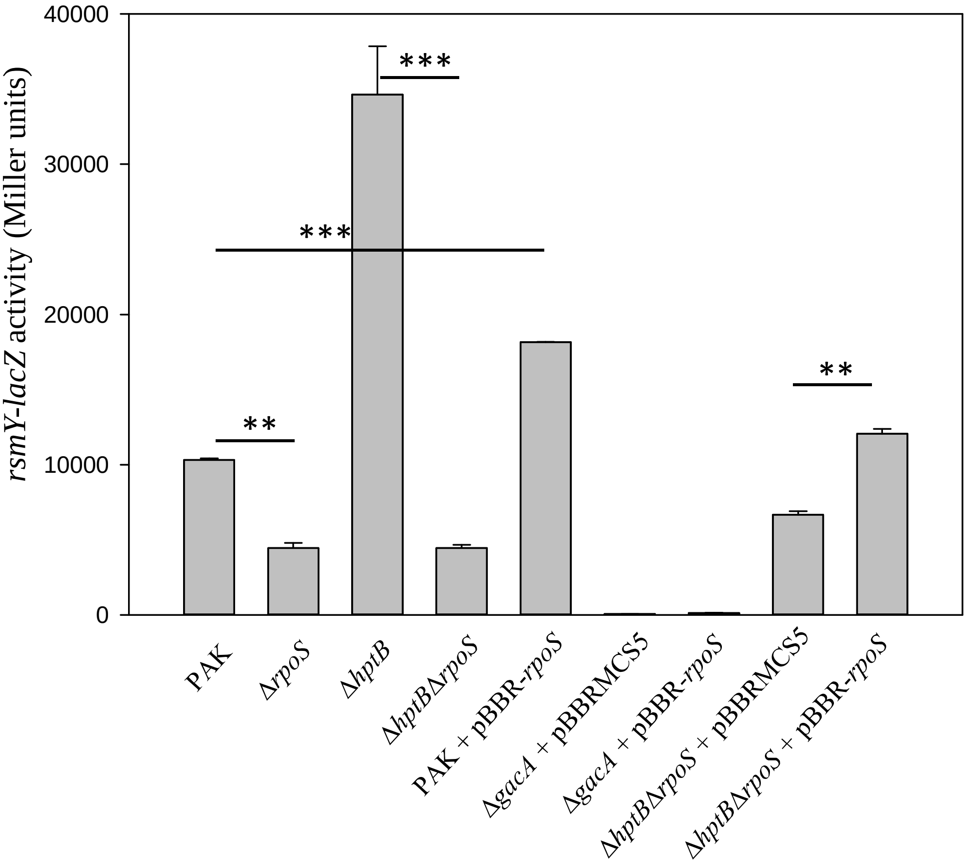

6H

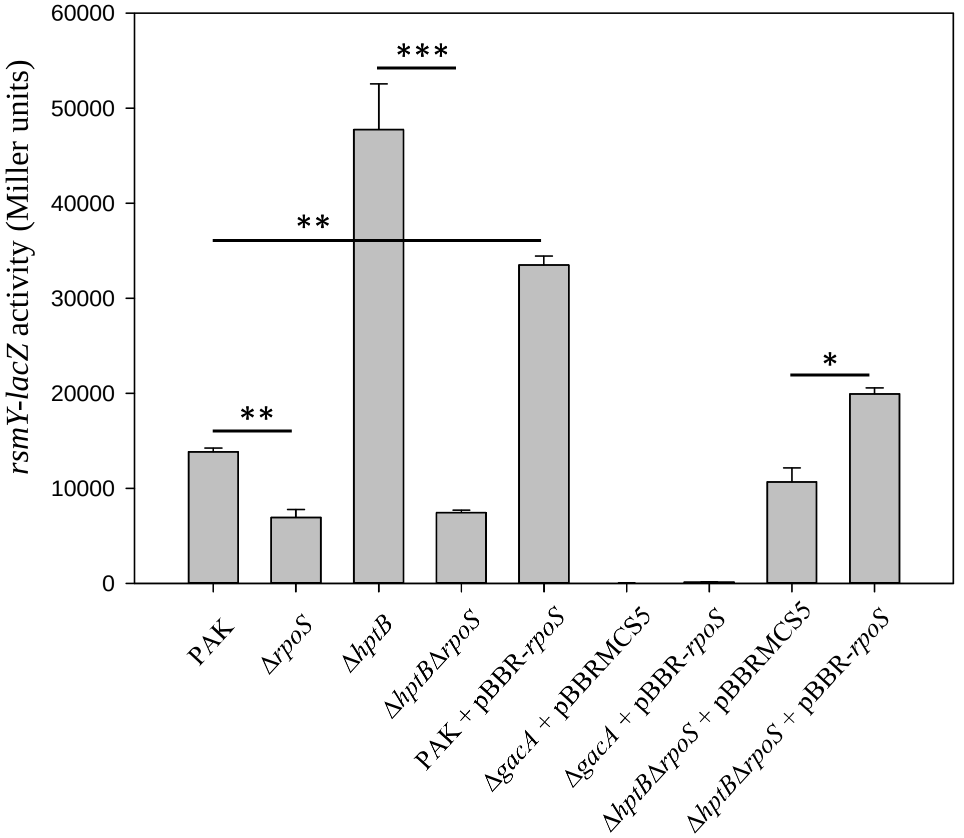

8H

Figure S4

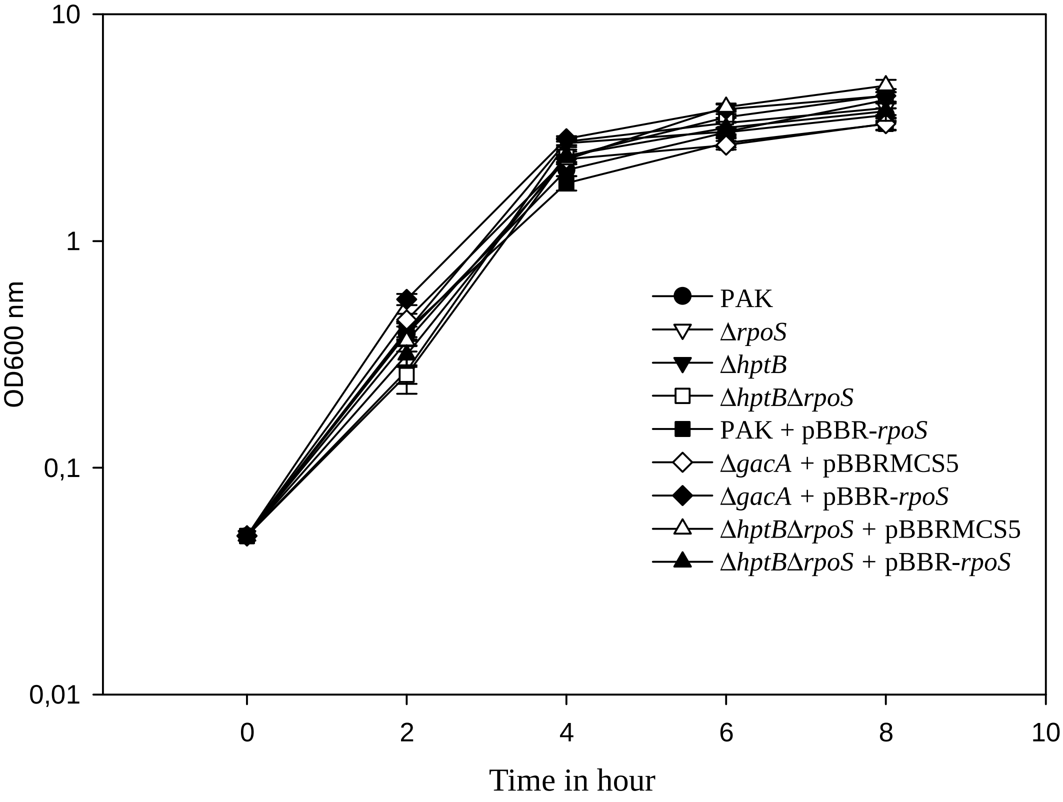

10
1
0,1
0,01
OD600 nm
0
2
4
6
8
10
Time in hour
PAK
ΔrpoS
ΔhptB
ΔhptBΔrpoS
PAK + pBBR-rpoS
ΔgacA + pBBRMCS5
ΔgacA + pBBR-rpoS
ΔhptBΔrpoS + pBBRMCS5
ΔhptBΔrpoS + pBBR-rpoS

Figure S5

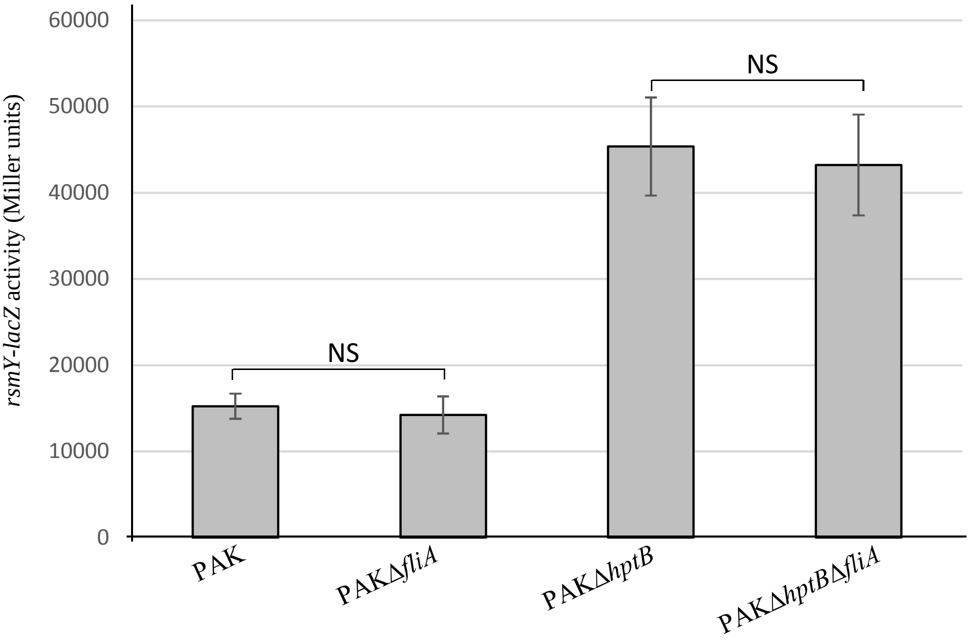

60000
50000
40000
30000
20000
10000
0
rsmY-lacZ activity (Miller units)
NS
NS
PAK
PAKΔfliA
PAKΔhptB
PAKΔhptBΔfliA

Figure S6

A.

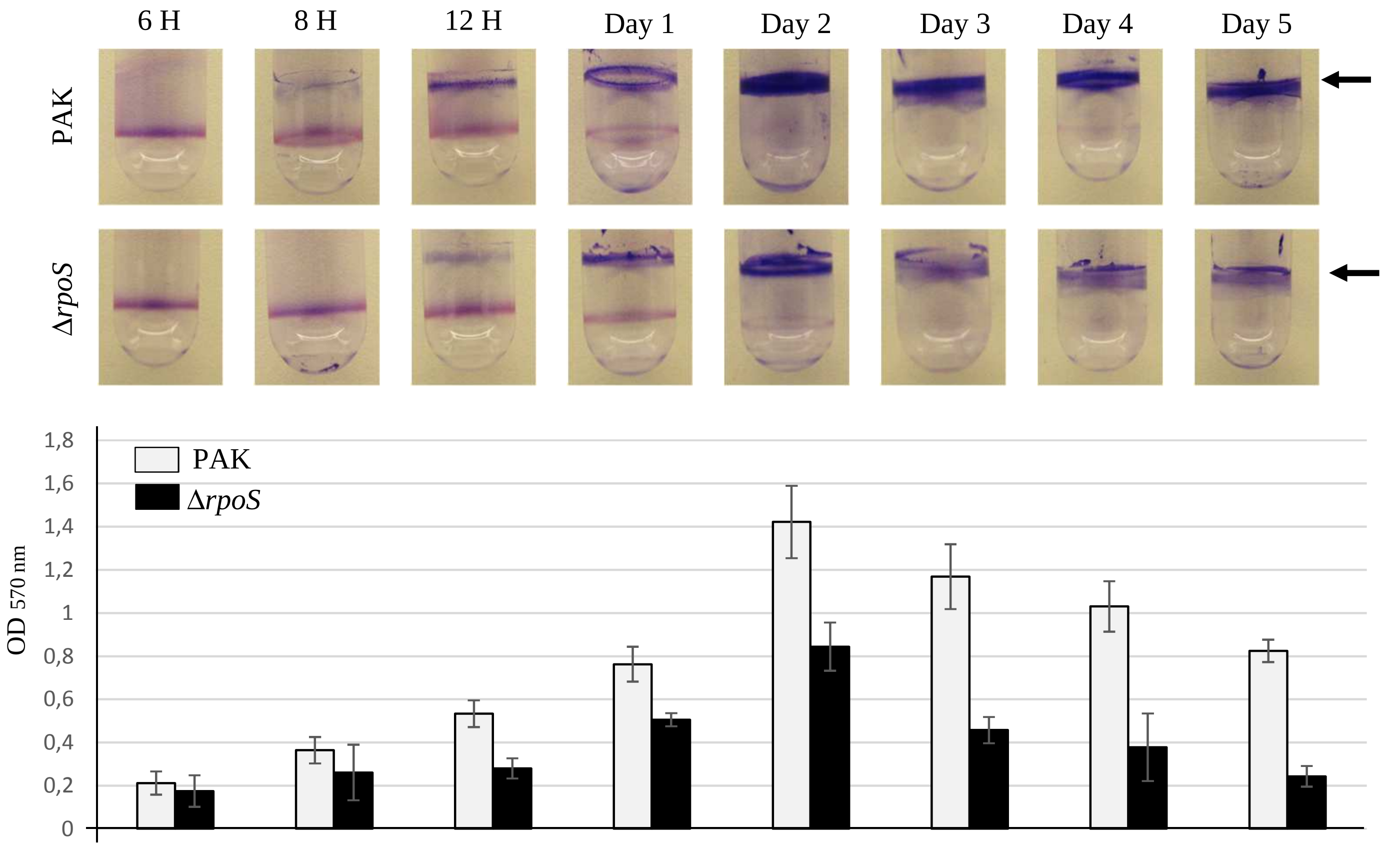
6 H
8 H
12 H
Day 1
Day 2
Day 3
Day 4
Day 5
PAK
ΔrpoS
1,8
1,6
1,4
1,2
1
0,8
0,6
0,4
0,2
0
OD 570 nm
PAK
ΔrpoS

B.

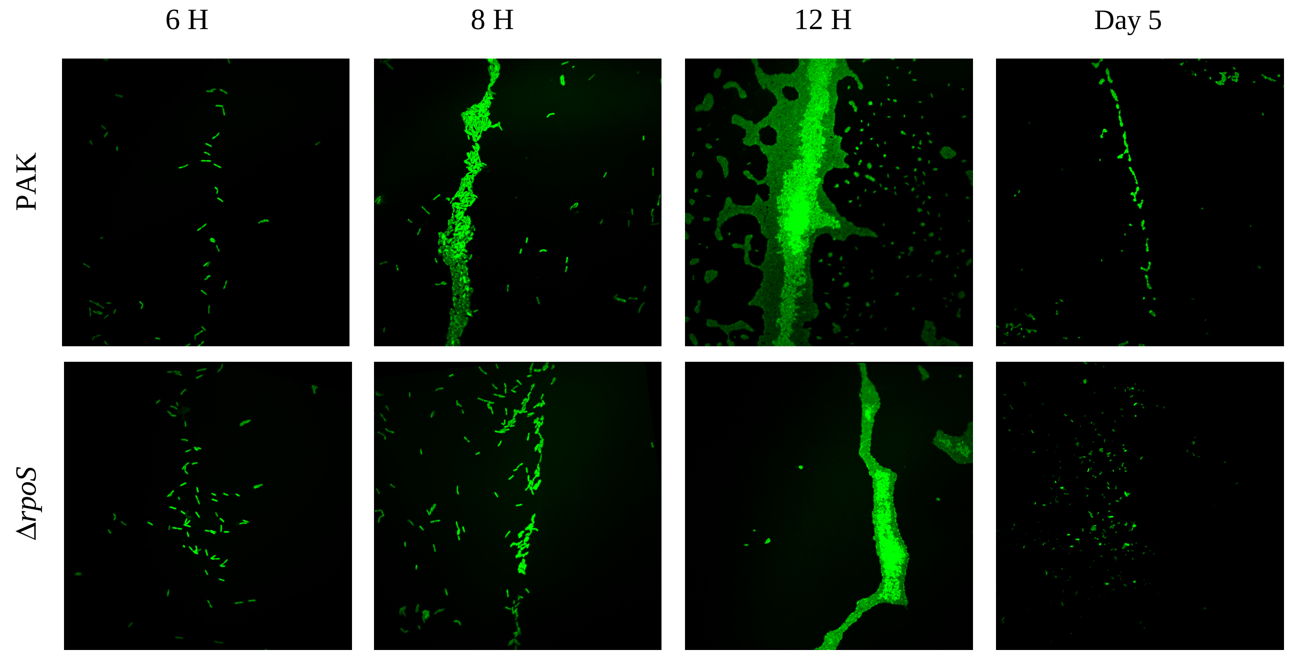
6 H
8 H
12 H
Day 5
PAK
ΔrpoS
